# The Digestive System of the *Arctocephalus australis* in Comparison to the Dog as a Land-Carnivore Model

**DOI:** 10.3390/ani12131634

**Published:** 2022-06-25

**Authors:** Rosario Martín-Orti, Carlos Tostado-Marcos, Juan-Pablo Loureiro, Ignacio Molpeceres-Diego, Enrique Tendillo-Domínguez, Inmaculada Santos-Álvarez, Pilar Pérez-Lloret, Juncal González-Soriano

**Affiliations:** 1 Departamento de Anatomía y Embriología, Sección Departamental de Anatomía y Embriología (Veterinaria), Facultad de Veterinaria, Universidad Complutense, Avenida Puerta de Hierro s/n, 28040 Madrid, Spain; rosamart@ucm.es (R.M.-O.); inmasant@ucm.es (I.S.-Á.); pilper01@ucm.es (P.P.-L.); 2 Fundación Mundo Marino. Av. X 157, San Clemente del Tuyú B7105, Provincia de Buenos Aires, Argentina; tostadomarcoscarlos@gmail.com (C.T.-M.); juanploureiro@gmail.com (J.-P.L.); ignamolp11@gmail.com (I.M.-D.); enriquetd95@gmail.com (E.T.-D.)

**Keywords:** *Arctocephalus australis*, anatomy, digestive system, adaptations

## Abstract

**Simple Summary:**

Marine mammals are warm-blooded vertebrates that behave in the same way as land mammals do but they spend most or all of their lives in the ocean. There is much previous research on whales, dolphins, or even different types of seals, including their behavior, health, anatomy or perception. Between all these fields, it is commonly accepted that anatomy is considered critical to understanding many physiological adaptations. For example, their ability to dive by holding their breath underwater for long periods of time. During this process they can postpone digestion for several hours. However, and contrary to what might be expected, our results show that being a carnivore seems to be the critical characteristic defining their digestive apparatus, the adaptations to the aquatic environment being less significant.

**Abstract:**

Marine mammals play a critical ecological role as both predator and prey. They are divided into three groups that share similar adaptations to their aquatic life, but that have very different origins and life patterns: sirenians, pinnipeds, cetaceans. The species object of our interest is the South American fur seal or *Arctocephalus australis*, a carnivore classified within the group of pinnipeds. The objective of the present study was to evaluate whether the anatomical characteristics of the *Arctocephalus australis’* digestive system are similar to that of other land-carnivores or if, on the contrary, this species shows anatomical adaptations related to their life in the ocean. The study was carried out on 11 cadavers of the species *Arctocephalus australis*, made up of two adults and nine juveniles, by means of the anatomical dissection of their entire isolated digestive system. We demonstrate that, with several exceptions, the anatomical characteristics of the digestive system of the *Arctocephalus australis* are similar to those in other carnivores. Therefore, our data constitute an important contribution for clinical diagnostic and conservation purposes, for both veterinarians and biologists.

## 1. Introduction

Conducting research into marine mammals is important in order to have a better understanding of their aquatic ecosystems. Pinnipeds, cetaceans and sirenians are mammals that develop their life in the marine aquatic environment, thanks to the large number of anatomical and physiological adaptations they have developed. A part of the life cycle of pinnipeds, such as feeding or exploration, takes place in the water, while another part, usually linked to periods of reproduction, hair shedding and rest, takes place on land. Instead, the life cycle of sirenians and cetaceans is completely aquatic [1,2,3,4,5,6].

The species object of our interest is the South American fur seal or *Arctocephalus* *australis*, a carnivore classified within the group of pinnipeds. Previous studies on these pinnipeds are numerous and focused on very different fields [7,8,9,10,11,12,13,14,15]. Yet, although anatomy is considered critical to understanding fundamental processes such as certain physiological adaptations, bibliography on the anatomy of the South American fur seal is scarce [5,16,17,18].

One of these adaptations in marine mammals (including the *Arctocephalus australis*) is their ability to postpone digestion for several hours while they are diving. Thanks to this process, it is possible to prevent oxygen consumption and instead, improve the oxygenation of muscles and vital organs, to extend the diving time safely [19,20,21,22,23,24].

The question that arises is whether this ability or other physiological adaptations modify the anatomical characteristics of the digestive system of the *Arctocephalus australis*, or if, on the other hand, it has the same general characteristics of the digestive system of other carnivores [25,26,27,28].

In the present study, we try to answer this question and thus, our main goal is to carry out an anatomical, systematic, and descriptive study of the South American fur seal’s digestive system, in comparison with the digestive apparatus of the dog, as this species could be considered as a carnivore model.

## 2. Materials and Methods

The anatomical study was carried out on 11 cadavers of the species *Arctocephalus australis*, made up of 2 adults and 9 juveniles. In all cases, the animals were found stranded on the coast, and taken to the Centro de Rescate y Rehabilitación Fundación Mundo Marino (FMM), where they died. The individuals were never euthanized. In all cases, they were frozen and preserved until their dissection in the necropsy room. Thawing time was at least 24–48 h. The animals were placed in dorsal recumbency. All dissections were carried out by veterinarians.

As a first step, the skin, blubber, and muscle must first be removed. With a scalpel blade, we made a first incision, under the chin, near the apex of the throat, and continued down the midline to the anus. If the animal was a male, we made the cut slightly left of the penile opening. The cut incised down to the blubber/muscle interface, without penetrating the skeletal muscle. Sometimes, we made perpendicular incisions on either side of the midline incision, creating a series of panels down the length of the animal. The blubber was removed by cutting through the fascia plane and reflecting the sections of skin and blubber away from the body, in a ventral to dorsal manner.

For the internal examination, we removed both front flippers, by pulling the appendage away from the body, and carefully cutting through the tissue that connects the flipper and scapula to the body wall. The same technique was carried out on the hip joints, sectioning the inguinal muscles, and displacing the femurs’ heads.

To expose the abdominal organs, we incised the abdominal wall from the last rib mid-ventral to the hip. To expose the internal tissues, we extended the most cranial cut laterally along the thoracic arch, reflecting the abdominal musculature.

Before cutting the ribs, to check the thoracic pressure, we punctured the diaphragm with a scalpel or scissors. To open the thoracic cavity, we removed the rib cage by cutting through each thoracic rib mid-articulation, which is the cartilaginous flex point that allows movement of the rib cage during inhalation and exhalation. Then, the rib cage was removed, and the thoracic cavity exposed.

To keep the esophagus intact, we removed the superficial musculature of the neck area, exposing the trachea and esophagus. Then, we made a triangular incision along the inner sides of the jaws. The hyoid apparatus was cut, and the tongue was extracted through the intermandibular space. Then, the cardiorespiratory system was progressively separated.

To make the esophagus independent, its connections with the respiratory system (esophagus–trachea) were removed. On the other hand, the esophagus was sectioned at the beginning, exactly at the level of the pharynx. To maintain the esophagus–stomach union, an eyelet-shaped cut was made around the esophageal hiatus. In the abdominal cavity, all adhesions with the omentum, as well as the junctions with the uro-genital apparatus, were detached. Finally, the entire digestive system was externalized by sectioning the most caudal part of the rectum, including the liver and the pancreas.

As mentioned before, the present study included different specimens: 9 juveniles and 2 adults, obviously each with a different degree of development. Because of this, the corresponding arithmetic means of the data obtained were calculated separately, to avoid deviations and erroneous data.

The anatomical data obtained from dissection were completed with a radiological analysis of the main digestive viscera. The radiographs were provided by FMM. All proceeded from animals that needed X-rays for diagnostic purposes. The X-ray equipment used for this purpose was a portable brand poskom 40 ma/90 kv. The visualizer was a FujiFilm model devo II.

## 3. Results

### 3.1. Oral Cavity

**Figure 1:**
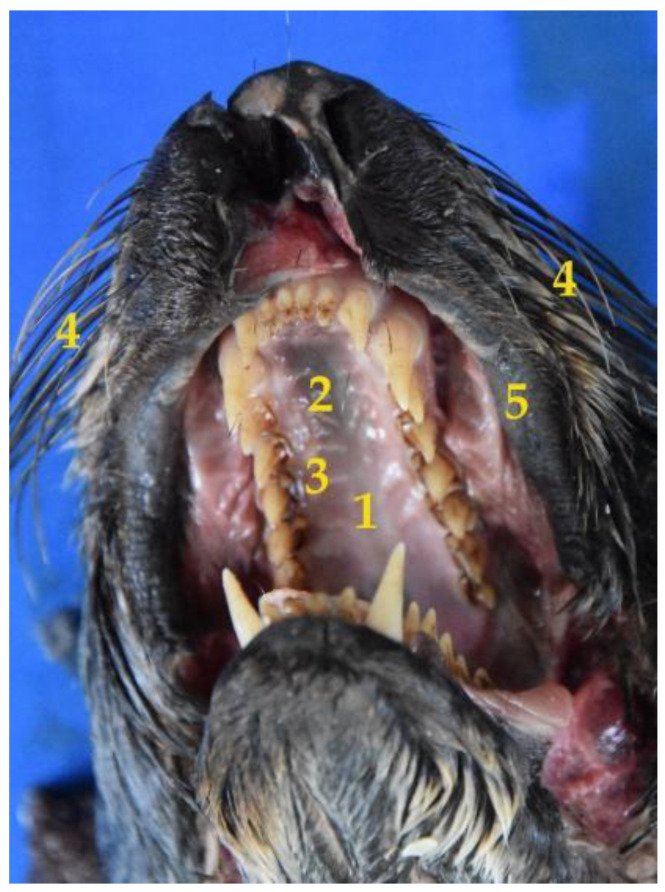
Image of the snout and the oral cavity of the *Arctocephalus australis.* (**1**) Hard palate; (**2**) median raphe; (**3**) curved ridges; (**4**) vibrissae; (**5**) upper lip.

The South American fur seal has an elongated and narrow oral cavity, with a wide mouth opening (Figure 1). The lips are thin and mobile with no tactile hairs. Instead, they have vibrissae in the muzzle, which are functional when diving. The upper lip has a little cleft (not very evident) in the central area, which divides it into two equal parts. In addition, the lateral edges of the lower lip are firm (Figure 1). The hard palate is made up of the palatine, maxillary and incisor bones. It is narrow in the rostral area, and widens caudally. The median palatine raphe divides the palate into two symmetrical halves, with a palatine raphe which is practically inconspicuous. There are also six to seven palatine ridges, curved and poorly marked extending on both sides of the palate. They never reach the median raphe. Caudally to the incisive teeth, there is a rounded shaped papilla: the incisive papilla. The mucosa may be slightly or not at all pigmented.

**Figure 2:**
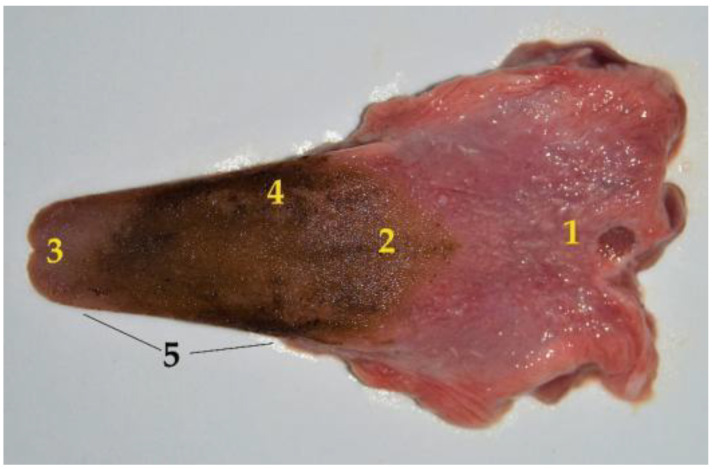
Dorsal surface of the South American fur seal tongue isolated from the oral cavity: (**1**) base; (**2**) body; (**3**) vertex (blunt and slightly bifid); (**4**) black coloration; (**5**) tongue ridge.

Something that is common and striking in the South American fur seals is the presence of a blackish coloration covering the surface of the teeth and the dorsal surface of the tongue, which is elongated and very mobile. It is wide at the base, although it progressively thins in a rostral direction, until it ends in a blunt and slightly bifid vertex. The tongue consists of three parts: base, body, vertex. No dividing mid-groove is observed on the dorsal surface (Figure 2). Some of the morphometric characteristics of the tongue are reflected in Table 1.

#### Teeth

An important anatomical feature is the dental formula:

*Arctocephalus australis*: 2 (I 3/2 C 1/1 P 4/4 M 2/1) = 36 (1)

There are characteristics that are worth highlighting. First, the morphological differentiation between premolars and molars in the South American fur seal has disappeared. Thus, the *Arctocephalus australis* has four jaw teeth and two mandibular teeth

**Figure 3:**
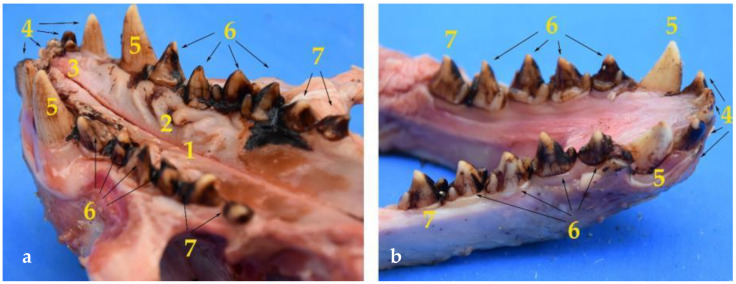
Images of the palate and teeth of the maxilla (**a**) and the jaw (**b**), which are composed of: (**1**) Hard palate; (**2**) palatine ridges; (**3**) incisive papilla; (**4**) incisors; (**5**) canines; (**6**) premolars; (**7**) molars. Additionally, the images show the substantia nigra adhered to the teeth surface.

It is interesting to remark that the crown of these dental pieces in the *Arctocephalus australis* is tritubercular, with the central prominence clearly larger than the laterals (Figure 3).

Concerning the incisors, the upper dental arch has a total of six, three on each side (Figure 3). The four centrals are bitubercular, both prominences being of identical size. The third incisor on each side (more lateral) has a completely different shape, as its size and appearance are similar to that of a canine. The crown has a single tubercle, is conical and curved. It is wide in the root, reducing its size progressively until ending in a sharp vertex. It could resemble a second, somewhat less developed, canine.

The mandibular incisors in the *Arctocephalus australis* number four in total, two on each side (Figure 3). Thus, the South American fur seal has lost two of its lower incisors.

### 3.2. Pharynx and Esophagus

The oropharynx has the typical characteristics of any carnivore. On the contrary, there are differences that affect the respiratory portion of the pharynx, which are not worth analyzing because the respiratory system is not the object of the present study.

**Figure 4:**
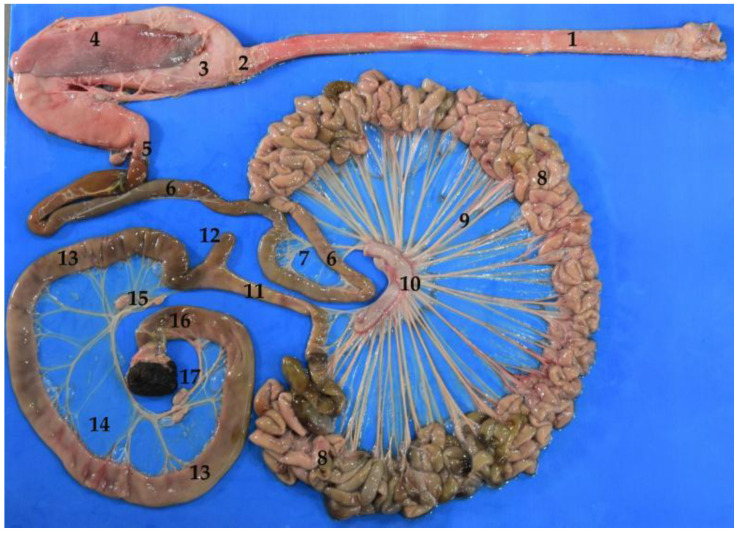
Isolated digestive system of the *Arctocephalus australis*. (**1**) Esophagus; (**2**) cardiac sphincter; (**3**) stomach; (**4**) spleen; (**5**) pylorus; (**6**) duodenum; (**7**) mesoduodenum; (**8**) jejunum; (**9**) mesojejunum and mesenteric vessels; (**10**) small intestine mesenteric lymphnodes; (**11**) ileum; (**12**) cecum; (**13**) colon; (**14**) mesocolon and mesenteric vessels; (**15**) large intestine mesenteric lymphnodes; (**16**) rectum; (**17**) anal canal.

The esophagus looks similar to that of other carnivores (Figure 4), although it is particularly wide and distensible, to avoid obstructions or different potential tissue damages when swallowing big prey. The esophageal width is constant along its course, except when approaching the cardiac sphincter, where a slight widening occurs. It has a great length, as in adults the average is 61 cm (Table 2).

**Figure 5:**
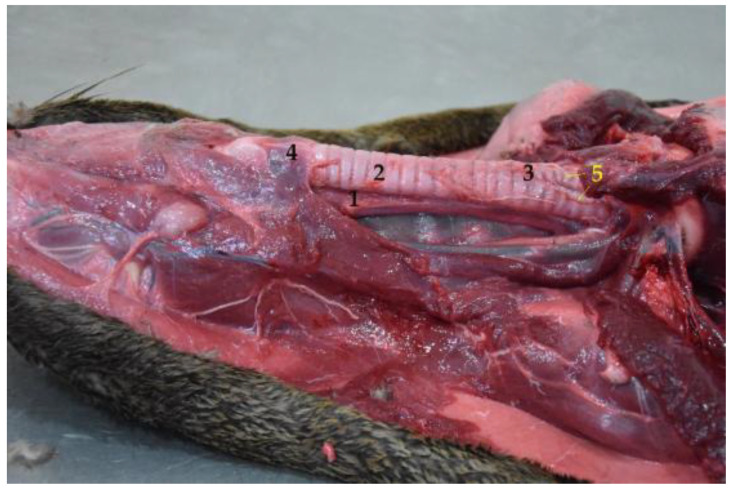
Cervical region with esophagus and trachea in situ (body in dorsal recumbency). Note the dorsal position of the esophagus in relation to the trachea. (**1**) Esophagus; (**2**) trachea; (**3**) tracheal bifurcation in the cervical region; (**4**) larynx; (**5**) bronchi.

Three esophageal parts are considered: cervical, thoracic and abdominal. The cervical part runs initially dorsal to the trachea (Figure 5). However, at the level of the caudal part of the neck, it becomes lateral and has a dorsolateral position with respect to the trachea, which in the *Arctocephalus australis* bifurcates at the thorax entrance, to become the thoracic esophagus. The esophagus position remains constant in relation to the bronchi (Figure 5), and ventral to the longus colli muscle, up to the third intercostal space and the aortic arch. From this point, it runs slightly tilted to the left, between the pulmonary lobes, until the diaphragm, passing through the esophageal hiatus, joining the stomach to form the abdominal esophagus.

### 3.3. Stomach

**Figure 6:**
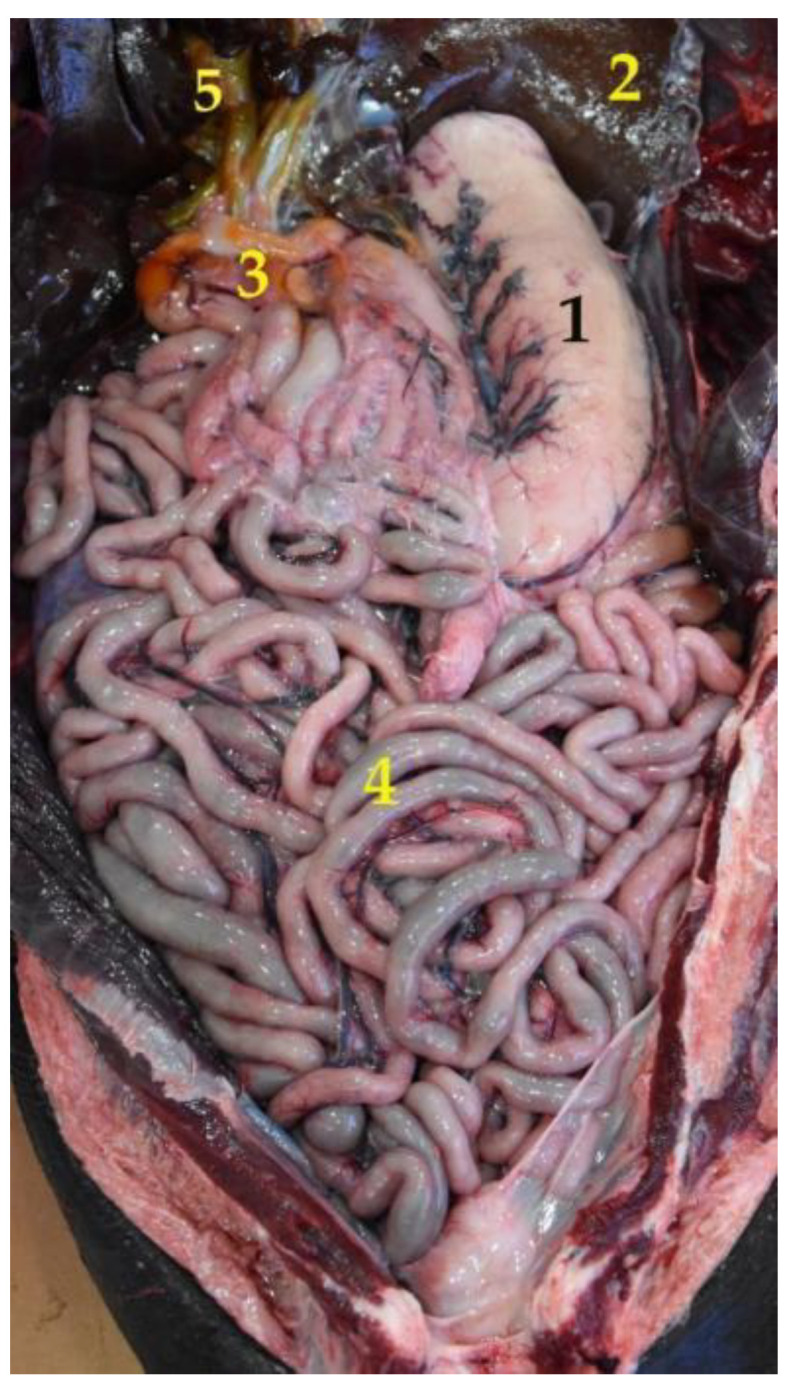
Topographical arrangement of the abdominal cavity (body in dorsal recumbency). The liver has been removed to see the gallbladder. (**1**) Stomach; (**2**) liver; (**3**) duodenum; (**4**) jejunum; (**5**) gallbladder.

**Figure 7:**
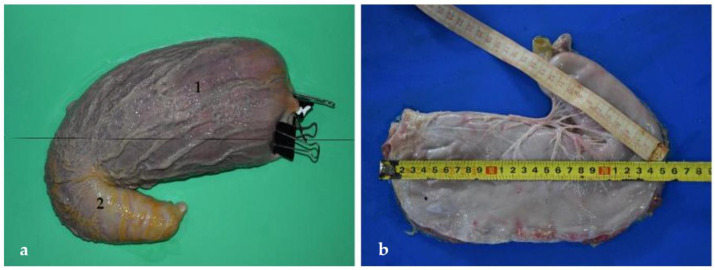
Stomach isolated from the abdominal cavity. (**a**) Anatomical pieces made up of expanded polyurethane (Fundación Mundo Marino) (**1**) Left part, (**2**) right or pyloric part.; (**b**) total length of the stomach or sum of the left and the pyloric parts (37 cm).

The stomach is a relatively large organ, which is located on the left side of the abdominal cavity. It is related to the liver craniodorsally and to the small intestine caudally (Figure 6), and is intimately related to the spleen (Figure 4). Laterally, it is in contact with the left abdominal wall, while ventrally it reaches the ventral abdominal wall, at the level of the xiphoid cartilage. It has two clearly differentiated parts: the left, comprising the cardiac part, the fundus and the body, and the right part, which is also called pyloric part (Figure 7a).

**Figure 8:**
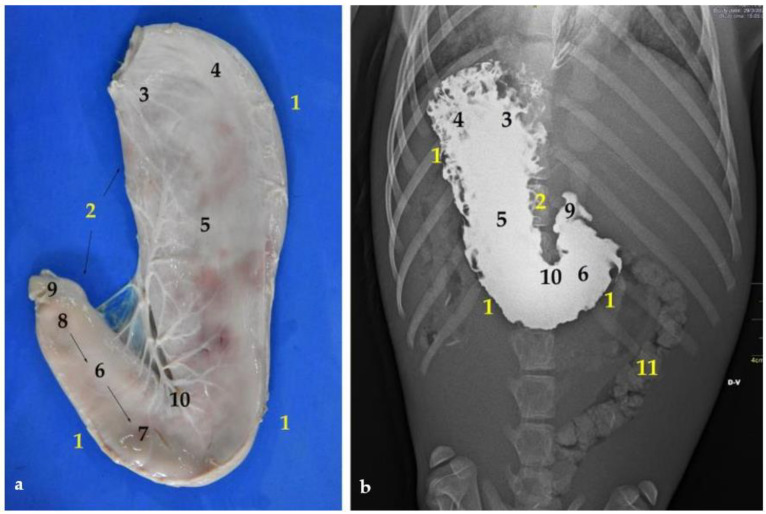
(**a**) Ventral view of the stomach; (**b**) dorsoventral contrast radiography. (**1**) Major curvature; (**2**) minor curvature; (**3**) cardiac part; (**4**) fundus; (**5**) body (**6**) right or pyloric part; (**7**) antrum pylorus; (**8**) pyloric canal; (**9**) pylorus; (**10**) angular incisure or angular notch; (**11**) colon.

**Figure 9:**
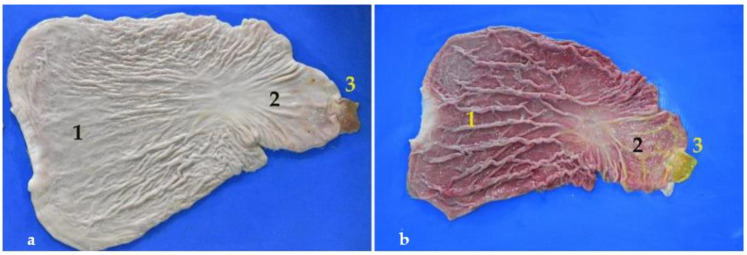
(**a**,**b**) Open stomach. Note the numerous folds of the mucosa. In (**a**) the stomach has been washed with water for days. (**1**) Fundic part; (**2**) pyloric part; (**3**) pylorus.

One of the relevant pieces of data included in the present study is the total length of the stomach, which is calculated by the addition of the left and right parts (Figure 7). The left is elongated and tubular shaped (Figure 8) and extends from the cardiac sphincter to the end of the stomach body. The right part, or pyloric part, is small and cylindrical in shape. It comprises the length from the end of the left part to the pylorus (Figure 8). According to the data obtained from the specimens of *Arctocephalus australis*, the mean average of the stomach length is 31 cm in juveniles and 53 cm in adults (Table 3). One of the most notable details of the stomach is the appearance of the mucosa, with numerous folds, which can be well appreciated thanks to an anatomical piece of expanded polyurethane, prepared according to the technique of De Sordi et al. [29] (Figure 7a and Figure 9). These anatomical pieces are also very useful for analyzing the stomach variation in size and its capacity of dilation when it is full of content. If the stomach is empty, the left part is strongly contracted but, as it progressively fills up, this left part expands widely. Curiously, the pyloric part (right), is much less affected by the intake and thus, its size is maintained whether the stomach is full or empty.

The stomach shows two curvatures: lesser and greater. First, the lesser curvature is straight and vertical, and forms the angular incisure, just at the bottom. This incisura is the consequence of a sudden change in direction of the pyloric part, which is directed cranio-dorsally (Figure 8). According to our results, the average length of the lesser curvature in juveniles is 20 cm, just half of that in adults, where it is 41 cm. On the opposite side is the greater curvature, which is considerably larger, with a length of 62 cm in adults and 38 cm in juveniles. Interestingly, in the South American fur seal, the greater curvature is not even double than the lesser (Table 3).

Another noteworthy fact is the stomach capacity, which in adults is of about 2–3 L and in juveniles it is of about 0.6–1 L.

### 3.4. Small Intestine

**Figure 10:**
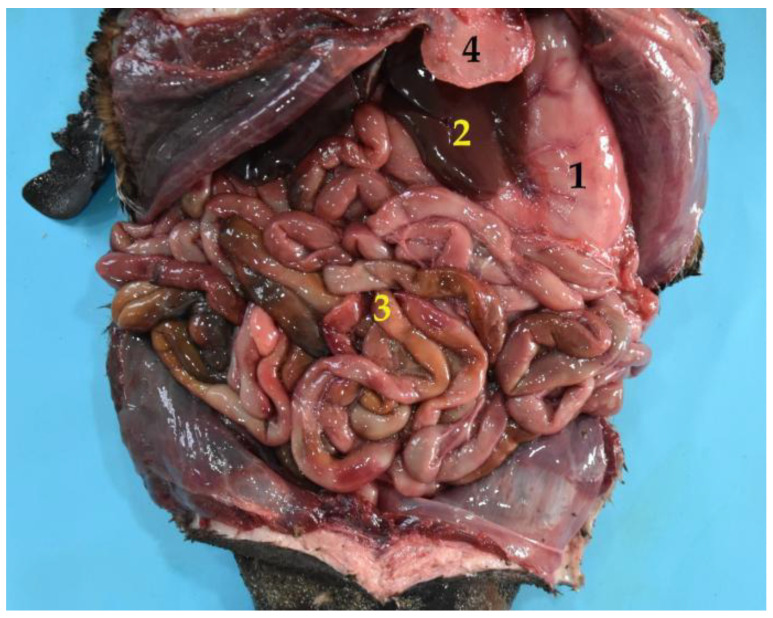
General view of the abdominal cavity (body in dorsal recumbency). (**1**) Stomach; (**2**) liver; (**3**) jejunum; (**4**) xiphoid cartilage.

The South American fur seal’s small intestine is peculiar, due to its significant large length. The shortest section is the ileum followed by the duodenum. The jejunum is the longest part, with an approximate length of 25 m in adults and between 12 and 15 m in juveniles (Table 4). Due to its size, the small intestine occupies a large part of the abdominal cavity (Figure 6 and Figure 10).

**Figure 11:**
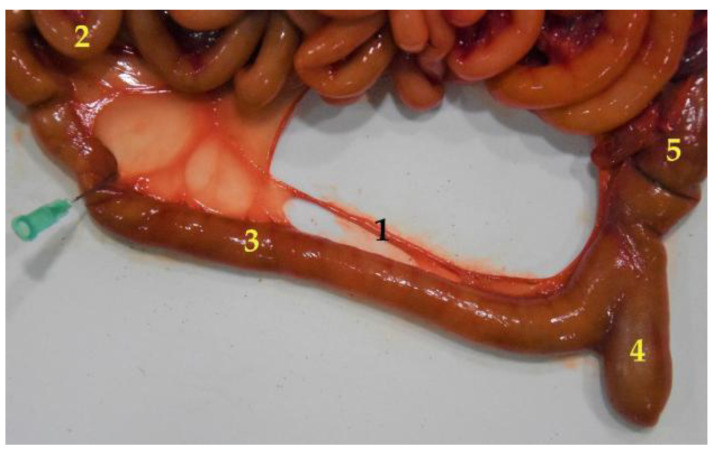
Differentiation of two segments of the small intestine (jejunum and ileum), pointed out by the last mesojejunal vessel, which splits off from the rest to reach the ileum. (**1**) Mesenteric vessel; (**2**) jejunum; (**3**) ileum; (**4**) cecum; (**5**) colon.

**Figure 12:**
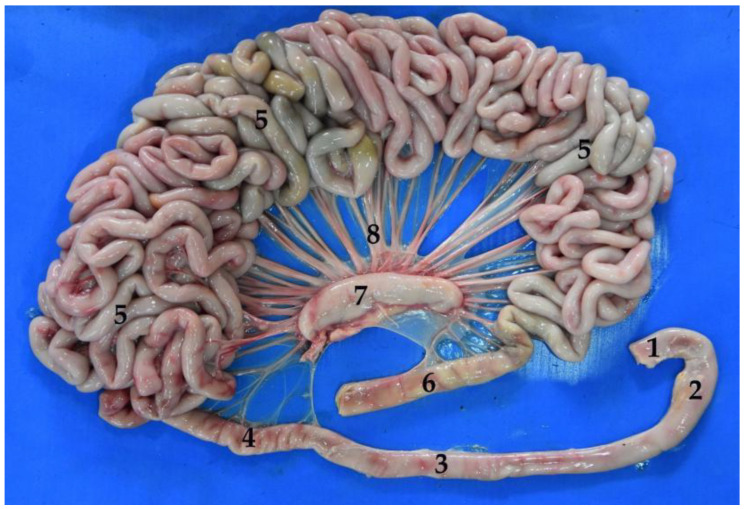
Isolated small intestine. Duodenum: (**1**) cranial part, (**2**) descending part, (**3**) transverse part, (**4**) ascending part; (**5**) jejunum; (**6**) ileum; (**7**) small intestine mesenteric lymph nodes; (**8**) mesenteric vessels.

To recognize the small intestine sections division, we used two criteria. First, the visualization of the mesenteric arteries, especially the last mesenteric artery, which runs isolated from the others and irrigates the ileum (instead of the jejunum), pointing out its beginning (Figure 11). Second, the different layout of the small intestine parts, which is striking. Thus, whereas the jejunum folds on itself and shows noticeable intestinal loops (Figure 4 and Figure 12), the ileum runs straight, while the duodenum is curved, without loops (Figure 12).

#### 3.4.1. Duodenum and Pancreas

**Figure 13:**
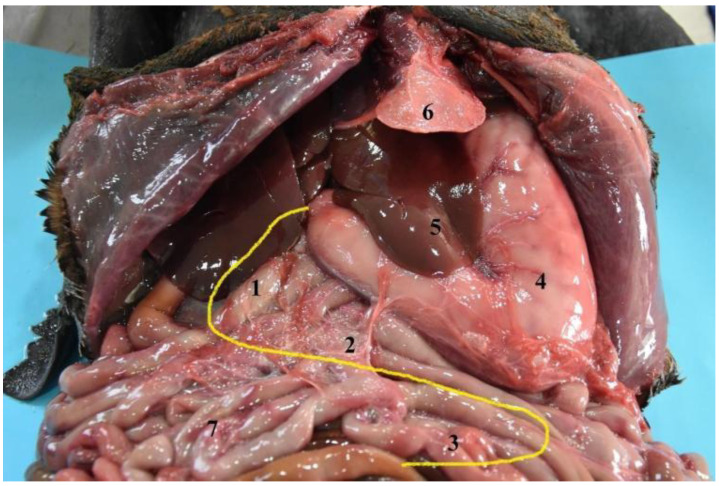
Topography of the duodenum in the abdominal cavity (body in dorsal recumbency). (**1**) Descending duodenum; (**2**) transverse duodenum; (**3**) ascending duodenum; (**4**) stomach; (**5**) liver; (**6**) xiphoid cartilage; (**7**) jejunum.

The duodenum is the first section of the small intestine, beginning at the pyloric sphincter. Initially, it runs caudodorsally, in relation to the visceral surface of the liver. Then, it continues caudally in contact with the right flank, until it turns medially from the right side to the left, at the level of the caudalmost part of the stomach, close to the xiphoid cartilage. Near the pelvis, it turns again medially and goes in relation to the ascending colon and the right kidney. Finally, the duodenum bends ventrally to join the jejunum (Figure 13).

**Figure 14:**
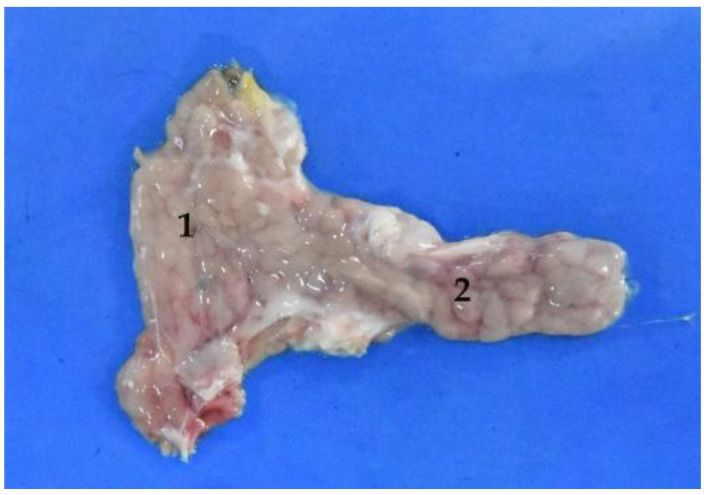
Isolated pancreas. (**1**) Right lobe; (**2**) left lobe.

**Figure 15:**
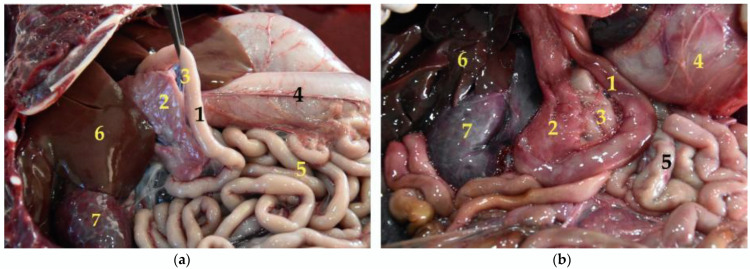
(**a**,**b**) Pancreas and duodenum in situ (body in dorsal recumbency). Note how they are intimately related. In (**b**) the stomach is displaced to visualize both viscera. (**1**) Duodenum; (**2**) pancreas; (**3**) mesoduodenum; (**4**) stomach; (**5**) jejunum; (**6**) liver; (**7**) right kidney.

**Figure 16:**
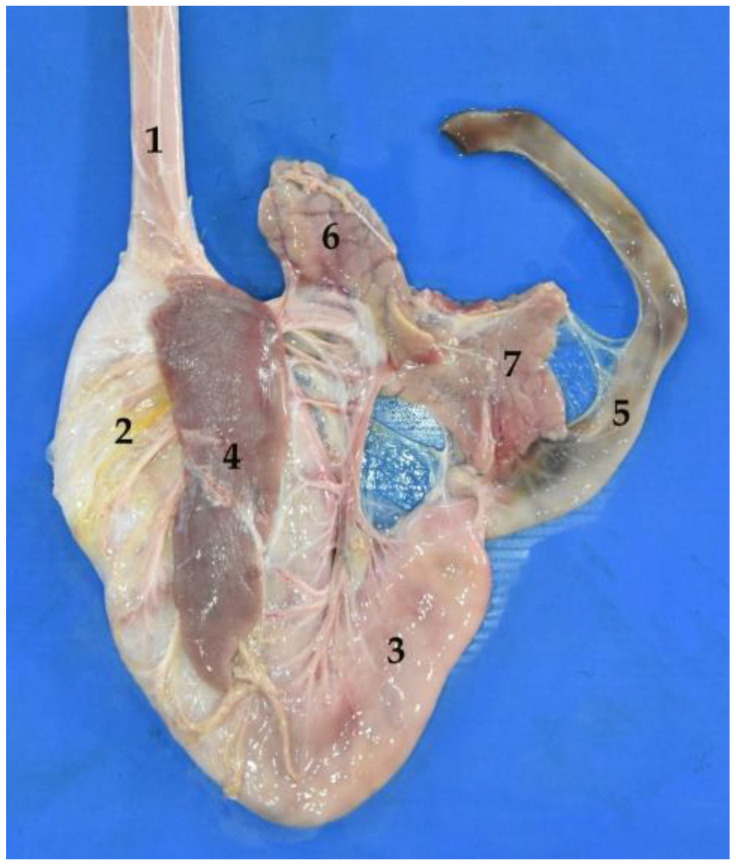
Partial view of the isolated digestive system. (**1**) Esophagus; (**2**) left part of the stomach; (**3**) pyloric part of the stomach; (**4**) spleen; **(5**) duodenum; (**6**) pancreatic left lobe; (**7**) pancreatic right lobe.

The pancreas is formed of two lobes, arranged in a V shape (Figure 14). The right lobe is closely related to the cranial part of the duodenum (Figure 15 and Figure 16). The left lobe is located between the stomach and the transverse colon. In the case of the *Arctocephalus australis*, this left lobe does not end in relation to the cranial pole of the left kidney.

#### 3.4.2. Jejunum

**Figure 17:**
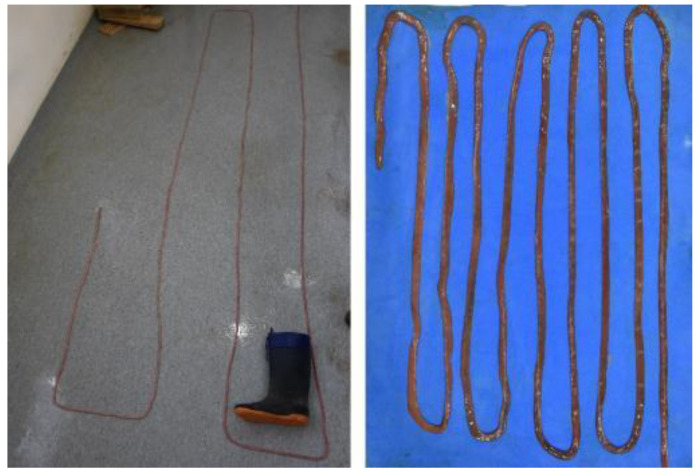
Two views of the extended jejunum after removing the mesojejunum. Note its great length.

As mentioned above, the jejunum is the longest section, with a length of 12–15 m in juvenile animals and 20–30 m in adults (Table 4 and Figure 17). It is located between the stomach, the liver, and the pelvis, occupying a great part of the abdominal cavity. The jejunum forms about 30 intestinal loops and maintains a constant width throughout its length.

#### 3.4.3. Ileum

**Figure 18:**
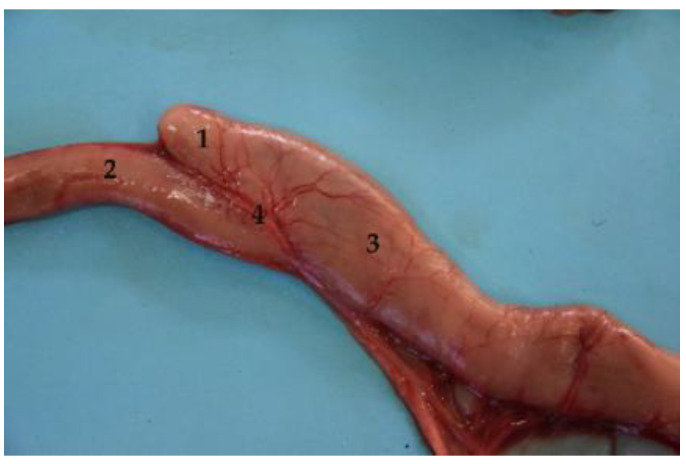
Anatomical relationship between ileum and cecum. (**1**) Cecum (**2**) ileum (final part); (**3**) beginning of the colon; (**4**) ileocecal union.

The ileum is the shortest section and represents the end of the small intestine. It is in the sublumbar region, joining the colon through the so-called ileocolic opening. The connection between ileum and cecum is close. However, it was not the same in all the specimens used in the present study. Thus, there are individual differences in the position, relationship and union between ileum, cecum, and colon, as it is practically parallel in some cases and perpendicular in others. (Figure 11 and Figure 18).

### 3.5. Large Intestine

The large intestine is composed of cecum, colon, rectum and anus. It does not show “teniae” or separate longitudinal ribbons of smooth muscle on the outside of the large intestine which give the colon its segmented appearance, or “haustra” (small pouches caused by sacculation). Regarding its total length, the mean of the adult South American fur seal large intestine was of 132.5 cm (Table 5). It is worth remarking that, in the case of the South American fur seal, there is a variation in the size between the small intestine segments (Table 4), and both colon and rectum (Table 5) have a significantly greater width in comparison with that of the small intestine.

#### 3.5.1. Cecum

**Figure 19:**
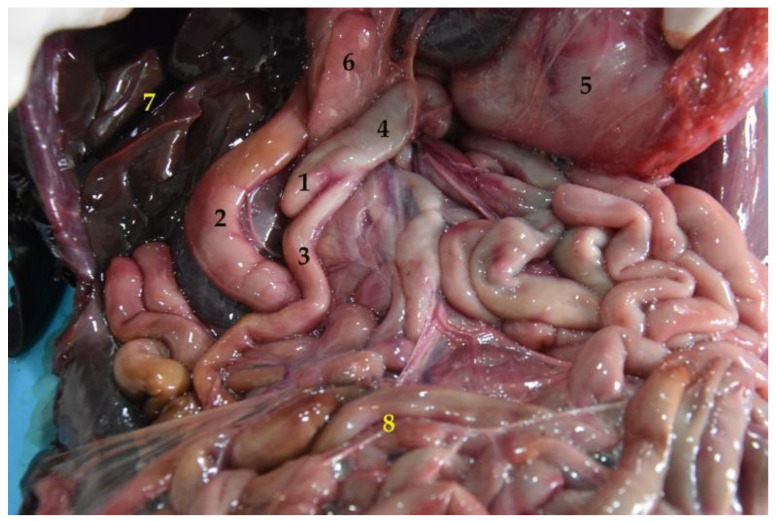
General view of the abdominal cavity (body in dorsal recumbency). The stomach is displaced to visualize the cecum in situ. (**1**) Cecum; (**2**) duodenum; (**3**) ileum; (**4**) colon; (**5**) stomach; (**6**) pancreas; (**7**) liver; (**8**) jejunum.

The cecum is located on the right side of the abdominal cavity, ventral to the pancreas and duodenum. One of its ends begins at the origin of the colon, lateral to the ileocolic orifice. The ileum only communicates with the colon, not with the cecum. So, the cecum could be described as a diverticulum in the proximal part of the colon, without any defined function. It communicates with the colon through the cecocolic orifice (Figure 18 and Figure 19).

The cecum of the South American fur seal is relatively small. In adults it measured 8.5 cm on average and 2.8 cm in juveniles (Table 5).

#### 3.5.2. Colon

**Figure 20:**
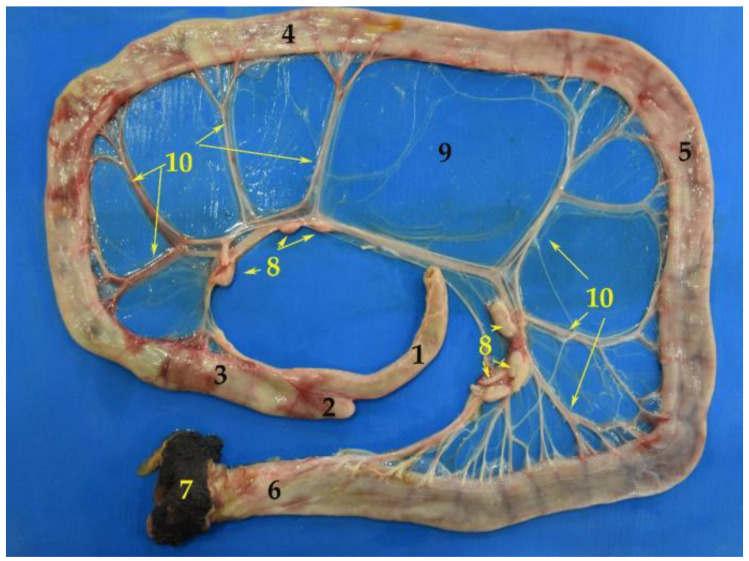
Image of the isolated large intestine. (**1**) Ileum; (**2**) cecum; (**3**) ascending colon; (**4**) transverse colon; (**5**) descending colon; (**6**) rectum; (**7**) anus; (**8**) lymph nodes; (**9**) mesocolon; (**10**) mesenteric vessels.

**Figure 21:**
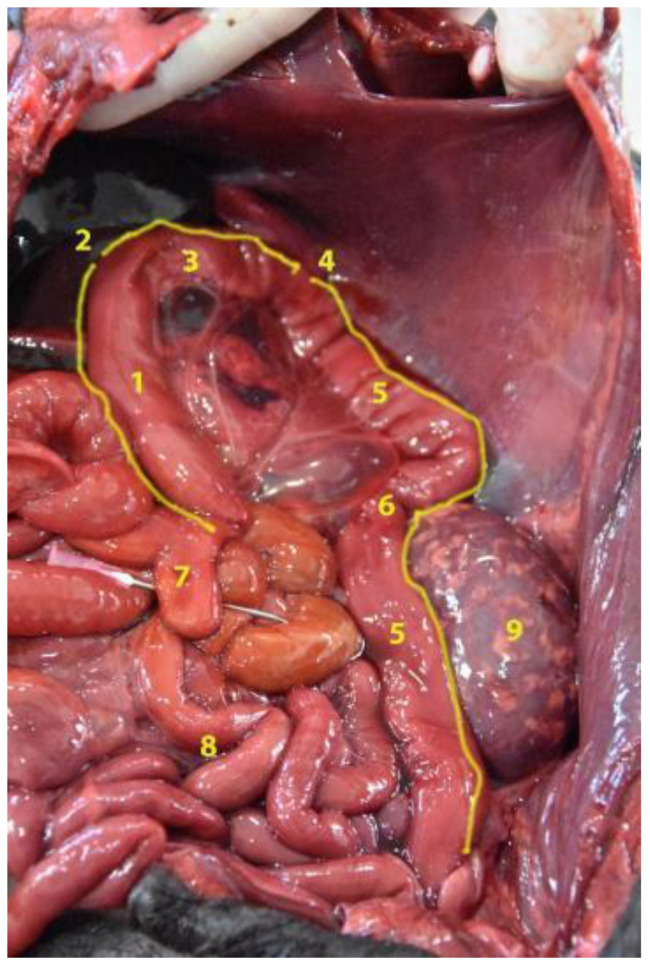
General view of the abdominal cavity (body in dorsal recumbency). Observe the colon with its different parts in situ. (**1**) Ascending colon; (**2**) right flexure of the colon; (**3**) transverse colon; (**4**) left flexure of the colon; (**5**) descending colon; (**6**) sigmoid of the colon; (**7**) cecum; (**8**) jejunum; (**9**) left kidney.

The colon is composed of three sections that run through the abdominal cavity. The first part corresponds to the ascending colon, followed by the transverse colon and, lastly, the descending colon (Figure 20 and Figure 21). It is attached to the sublumbar region by the mesocolon, which comes from the common mesentery. The ascending colon is short. It passes cranially along the medial surface of the duodenum and the right lobe of the pancreas, until it reaches the pyloric portion of the stomach. Then, the ascending colon changes direction to the left side and crosses the median plane. This section is known as the transverse colon. The last portion is the descending colon, which is located entirely on the left side. It passes caudally to the sublumbar region, along the medial border of the left kidney. Finally, it leans towards the median plane, conforming a “sigmoid” to start the rectum. It is possible to find isolated lymph nodes in both the first part and the final parts of the colon (Figure 20).

#### 3.5.3. Rectum

**Figure 22:**
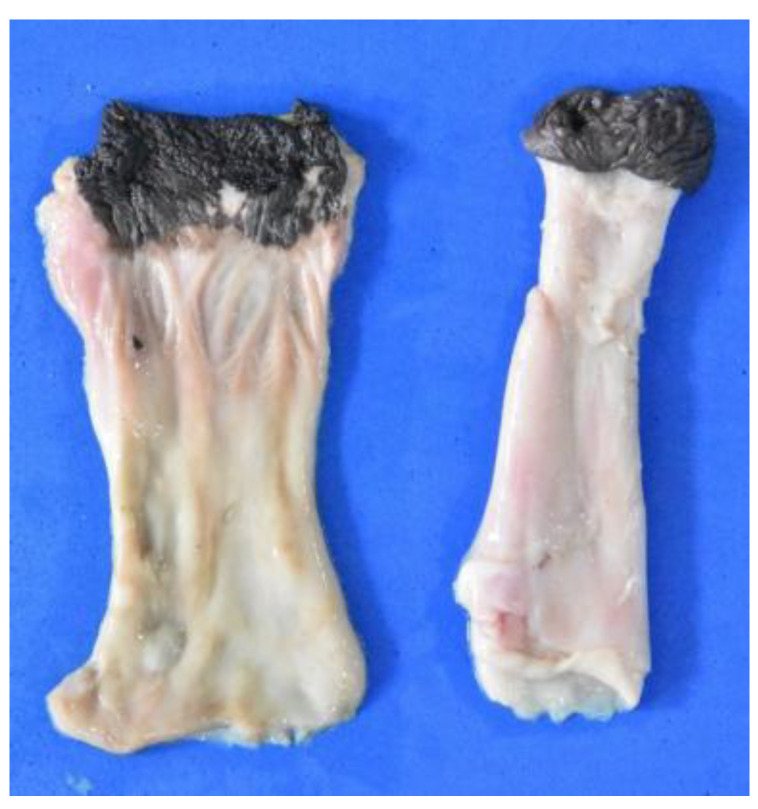
Image of two rectum of different specimens (the one on the left is opened).

The rectum is the final part of the digestive system (Figure 22). It is intrapelvic in terms of location and practically covered by the peritoneum. No lymph nodes are present in this digestive section.

### 3.6. Liver

This is the largest gland of the organism, as it represents 3% of the total weight. For example, in an adult South American fur seal (much bigger in size), the average reached 1000 g.

**Figure 23:**
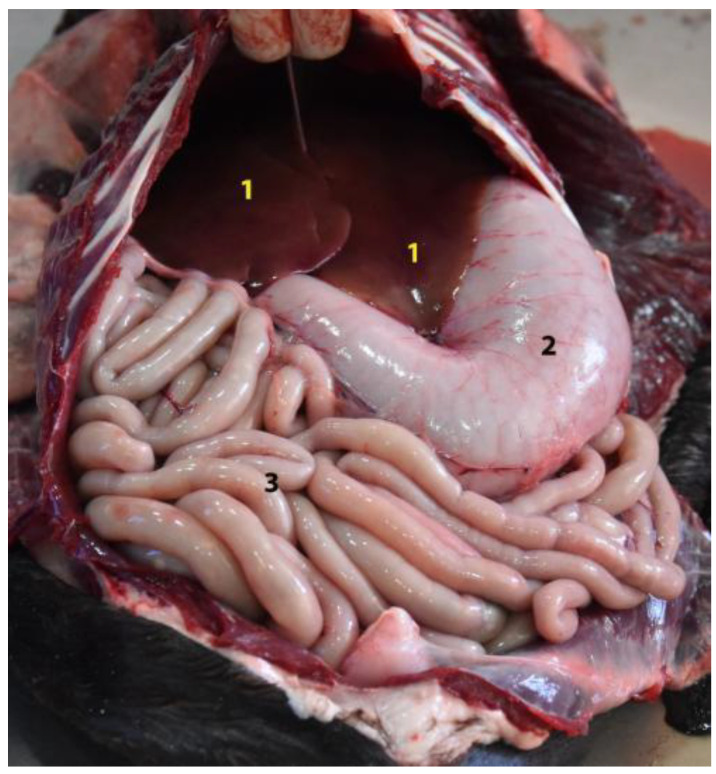
General view of the abdominal cavity (body in dorsal recumbency). Note the liver in situ. (**1**) Parietal surface of the liver; (**2**) stomach; (**3**) small intestine.

**Figure 24:**
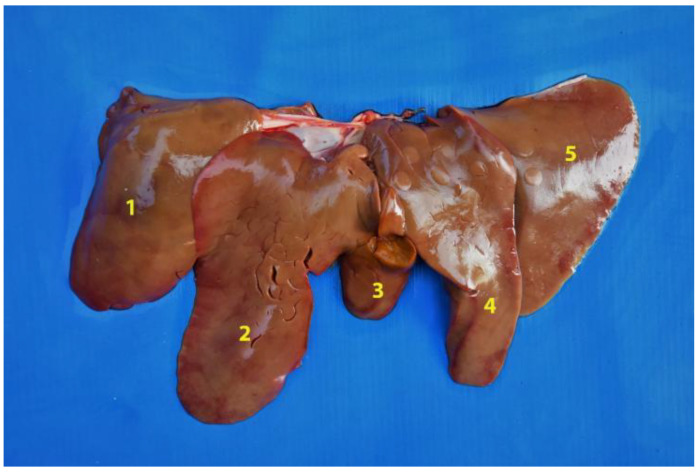
Diaphragmatic face of the liver. (**1**) Right lateral lobe; (**2**) right medial lobe; (**3**) quadrate lobe; (**4**) left medial lobe; (**5**) left lateral lobe.

**Figure 25:**
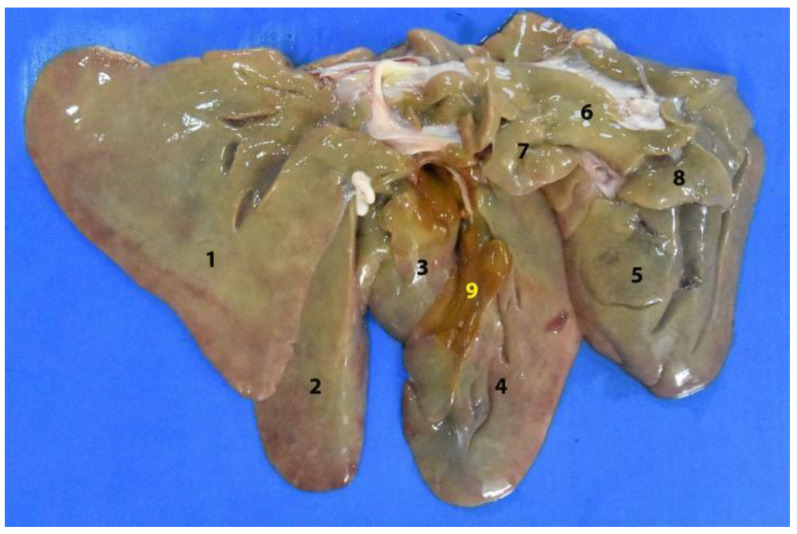
Visceral face of the liver. (**1**) Left lateral lobe; (**2**) left medial lobe; (**3**) quadrate lobe; (**4**) right medial lobe; (**5**) right lateral lobe; (**6**) caudate lobe; (**7**) papillary process; (**8**) caudate process; (**9**) gallbladder fossa.

**Figure 26:**
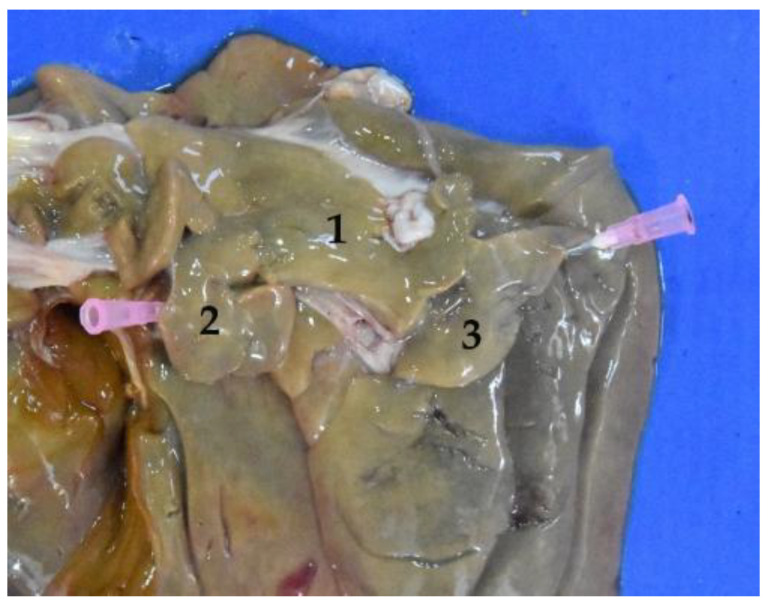
Visceral face of the liver. (**1**) Caudate lobe; (**2**) papillary process; (**3**) caudate process.

The liver is located in the intrathoracic portion of the abdominal cavity. It is divided into four main lobes: the quadrate lobe, the caudate lobe, the right lobe and the left lobe (Figure 23 and Figure 24). The right and left lobes are also subdivided, giving rise to a lateral and a medial lobe. On the visceral side, it is possible to observe the caudate lobe, which is made up of the caudate process on the right side, and the papillary process on the left (Figure 25 and Figure 26).

On the other hand, the liver is a relatively moldable organ, as it is easily adapted to the surrounding structures. The diaphragmatic or parietal surface is very convex. This surface fits perfectly with the concavity of the diaphragm and the ventral wall of the abdomen. On the visceral side, the hepatic lobes vary their configuration and are arranged irregularly, to have a perfect adaptation to the adjacent viscera. The stomach is located among them, especially because of its changing size, which depends on its degree of repletion.

### 3.7. Gallbladder

**Figure 27:**
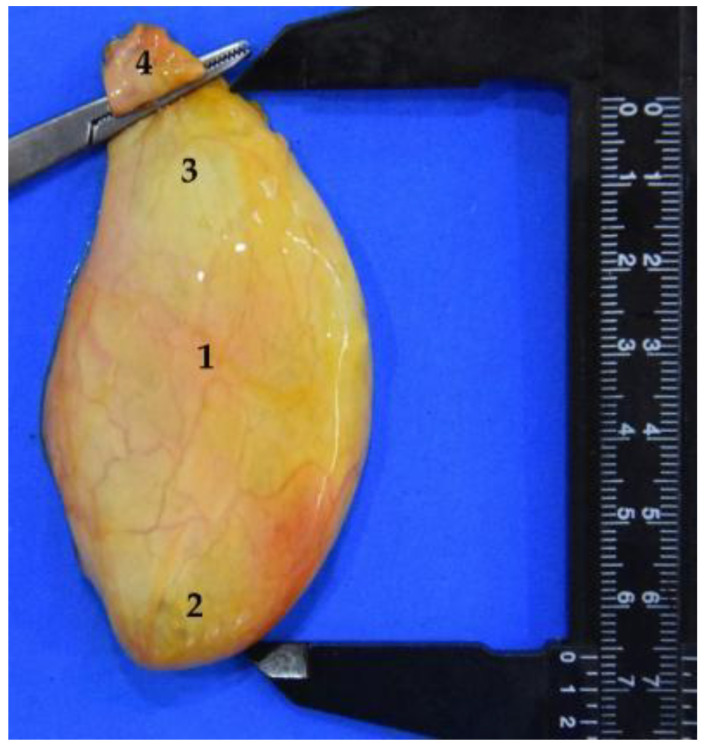
Isolated gallbladder. The total length can be observed, 6.5 cm. (**1**) Body; (**2**) fundus; (**3**) neck; (**4**) begining of the cystic duct.

The gallbladder is located in the gallbladder fossa, on the visceral face of the liver (to which it is firmly attached) and in relation to the quadrate lobe (Figure 27). It has three distinct parts, fundus, body and neck, which continues as a duct. No more anatomical features are worth mentioning.

### 3.8. Radiology

**Figure 28:**
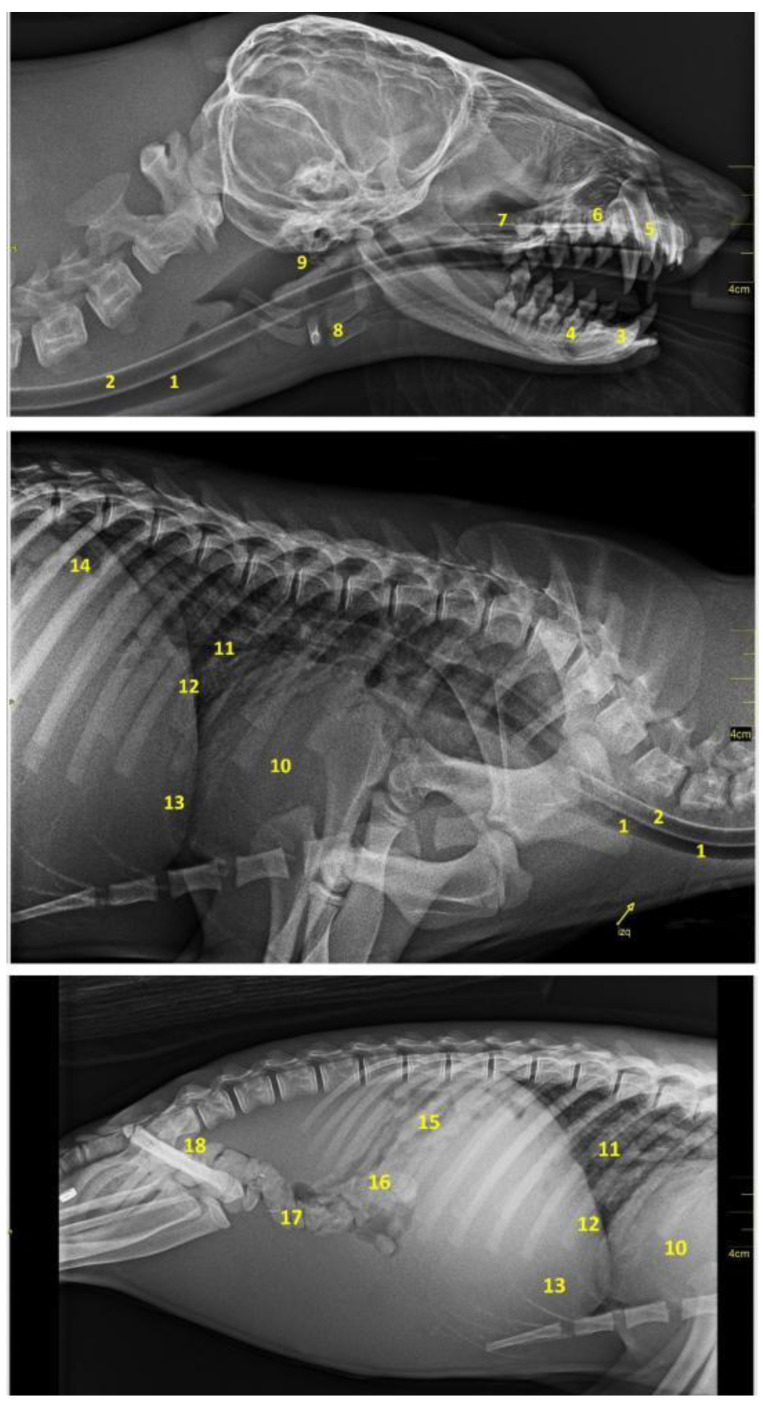
Radiographic images in laterolateral projection of the head, neck and cavities. (**1**) Trachea; (**2**) esophagus; (**3**) lower right canine; (**4**) lower right 2nd molar; (**5**) upper right canine; (**6**) maxillary right 2nd premolar; (**7**) maxillary; (**8**) hyoid; (**9**) pharynx; (**10**) cardiac profile; (**11**) bronchial tree; (**12**) diaphragmatic border; (**13**) liver; (**14**) stomach; (**15**) jejunal loops; (**16**) ascending colon; (**17**) transverse colon; (**18**) descending colon.

As mentioned, the anatomical description of the digestive system of the *Arctocephalus australis* was completed with a radiological study. Figure 28 shows the appearance of the main digestive viscera and its topographical relationships.

## 4. Discussion

As mentioned before, marine mammals play a critical environmental role, ensuring a balance in the ocean’s ecosystem. Although they live most of their lives in or very near the ocean, they have the typical characteristics of all mammals. For example, they breathe air through lungs, they are warm-blooded and they nurse their young. Surprisingly, however, the anatomy of many of these species is not well known. This is the case of the *Arctocephalus australis*, on which there are practically no scientific reports to explain its anatomy in detail. It is important to remark that the animal’s anatomy could justify certain physiological phenomena such as its ability to postpone digestion for several hours while they are diving. So, the question is: does the digestive system of *Arctocephalus australis* present adaptations that justify this physiological phenomenon? Or, on the contrary, is its digestive system similar to that of other land-carnivores, and not adapted to aquatic life? To answer this question, in the present paper we describe the digestive apparatus of the South American fur seal, to make a comparison to that of the dog, as a typical model of land-mammal.

As mentioned before in relation to the oral cavity, the upper lip has a little cleft (not very evident) in the central area, which divides it into two equal parts. In other carnivores, this cleft is much more marked, and is called the middle groove or filter [26]. The lateral edges of the lower lip are firm, while in dogs these edges are flaccid and denticulated [26].

In the *Arctocephalus australis*, the palatine raphe is almost inconspicuous, but in dogs, it forms a crest, as the palatine ridges transverse and are much more marked [25]. The incisive papilla is the same in both species [26]. Something curious to mention in the South American fur seal is the presence of a blackish coloration covering the surface of the teeth and the dorsal surface of the tongue. This striking detail is probably linked to their eating habits, and this does not exist in dogs.

The tongue consists of three parts: base, body, vertex. No dividing mid-groove is observed on the dorsal surface, contrary to what has been described in other carnivores [26]. Erdogan et al. [30] have shown that gustatory papillae on the tongue of pinnipeds are reduced in number and simplified in comparison to other carnivores; this and other factors may be responsible for the reduced or absent taste perception in these animals, in comparison with dogs.

In relation to teeth, the *Arctocephalus australis* shows important differential anatomical features in its dental formula, with variations that are worth highlighting. First, the morphological differentiation between premolars and molars in the South American fur seal has disappeared. This is probably because these teeth have currently come to have the same function. They have also decreased in number.

*Arctocephalus australis*: 2 (I 3/2 − C 1/1 − P 4/4 − M 2/1) = 36

Dog: 2 (I 3/3 − C 1/1 − P 4/4 − M 2/3) = 42

The dog has four jaws and six mandibular molars [31], whereas the *Arctocephalus australis* has four jaw teeth and two mandibular teeth, that is, four fewer mandibular teeth. This decrease could be linked to a progressive functionality loss of these dental pieces, as South American fur seals do not chew the food. They simply tear large prey before swallowing. This lack of use has probably caused its disappearance.

Although premolars and molars are similar in size and shape, there are clear differences concerning the number of roots. The first two upper and lower premolars have one root; the rest have two. In the case of dogs, there are three roots in the upper premolar four (carnassial molar tooth) and in the upper molars as well [31]. The crown of these dental pieces in the *Arctocephalus australis* is tritubercular, with the central prominence clearly larger than the laterals.

The mandibular incisors in the *Arctocephalus australis* number four in total, two on each side. Again, there are differences in comparison with the dog. This species presents three mandibular incisors on each side, which makes six in total. Thus, the South American fur seal has lost two of its lower incisors, probably because of its eating habits.

The esophagus looks similar to that of other carnivores. In comparison with dogs, there are two characteristics to remark upon, which could be related to the eating habits of the species. First, the esophageal length, as in adult South American fur seals, the average is 61 cm, which double that of a medium-sized dog (15–25 Kg), where it is about 30 cm [25]. Then, the esophagus of the *Arctocephalus australis* it is particularly wide and distensible, probably to avoid obstructions or different potential tissue damages when swallowing big prey.

The stomach of the South American fur seal is elongated and tubular shaped in contrast to that of other carnivores, such as dogs, where the stomach is piriform-shaped [26]. It is interesting to remark that in the South American fur seal, the greater curvature is not even double that of the lesser (Table 3), whereas in the dog, the greater curvature is almost four times larger than the lesser [26]. Another noteworthy fact is the stomach capacity, which is poor in comparison to other carnivores. Thus, in adults it is about 2–3 L and in juveniles it is about 0.6–1 L; this is much less than in dogs, where it is approximately of 5 L in individuals of an average size of 15–25 kg.

This small stomach of the South American fur seal remembers that in the horse, which is very small in comparison with its body size [25]. This is probably an indication that the capacity of feed storage, one of the stomach main functions, is poor in the Arctocephalus australis in comparison with other carnivores.

The South American fur seal’s small intestine is peculiar, due to its significant large length in comparison to other carnivores’ species. It would resemble the small intestine of herbivores, instead of that of dogs (33 m in horses or 31 m in small ruminants, whereas in dogs it is 3.6 m) [25]. Herbivores have a longer small intestine than carnivores because they eat plant- and grass-based foods that are high in cellulose, which takes a long time to digest. Although the *Arctocephalus australis* is a carnivore, its small intestine’s length could indicate that the nutrients will have more time to be absorbed into the body as they pass through. This is probably a different strategy of extracting food nutrients compared to that of other carnivores, including the dog.

The jejunum is the longest section, with a length of 12–15 m in juvenile animals and 20–30 m in adults (Table 4 and Figure 17). It is interesting to highlight its exaggerated length in comparison with other carnivore species, such as the dog (2.5 m) [25]. The jejunum forms about 30 intestinal loops, much more than in the case of the dog [26] and maintains a constant width throughout its length. As products of digestion (sugars, amino acids, and fatty acids) are absorbed into the bloodstream here, this long jejunal length indicates a great amount of surface area to optimize digestion and nutrient absorption in the *Arctocephalus australis*.

As in dogs, the large intestine of the South American fur seal does not show “teniae” or separate longitudinal ribbons of smooth muscle on the outside of the large intestine which give the colon its segmented appearance, or “haustra” (small pouches caused by sacculation). The mean total length of the adult South American fur seal’s large intestine was of 132.5 cm (Table 5), which is double that of the dog (60–75 cm) [26].

When compared, significant differences in the large intestine internal gauge of dogs and *Arctocephalus australis* were observed. In the dog, there is no variation between the small and the large intestines, as the internal diameter remains practically the same in all their parts [26]. On the contrary, in the case of the South American fur seal, there is a variation in size between the small intestine segments (Table 4 and Table 5), and both colon and rectum have a significantly greater width in comparison with that of the small intestine. This characteristic could mean a different way to absorb water and salts from the material that has not been digested.

As in the rest of non-marine carnivore species, the ileum only communicates with the colon, not with the cecum. As in the dog, the other end is blind and round-shaped [32].

The cecum of the South American fur seal is relatively small. In adults, it measured 8.5 cm on average, and it was 2.8 cm in juveniles (Table 5). These values are considerably lower than those measured for the dog, in which it can measure up to 15 cm [26]. This is probably due to the nature of their diet. The shape is also different, since in the *Arctocephalus australis* it is straight, while in the dog it is flexuous [26,32].

Concerning the colon, the arrangement is identical to that of other carnivores. As in the case of the dog, it is possible to find isolated lymph nodes in both the first part and the final parts of the colon (Figure 20) [25].

Finally, in relation to the rectum, no lymph nodes are present in this digestive section, contrary to what happens in the dog [32].

## 5. Conclusions

The anatomical and topographic features of South American fur seal’s digestive system are very similar to those presented by other non-marine carnivores, as is the case of the dog. However, the present study shows some specific anatomical differences with other non-marine carnivores. For instance, the changes described on the teeth formula, as a result of the fur seals’ adapted diet and their way of ingesting prey, without chewing. When studying adult seal individuals, we find that both the small and the large intestines double their length and weight with respect to their juvenile counterpart. However, surprisingly, in adults fur seals, the lumen of both intestines is only increased by 50%, so these parameters do not seem to be proportional. Compared to the dog, both intestines also show significant differences. The South American fur seal’s large intestine is double that of the dog, whereas its small intestine can be up to eight times longer. There is no evidence that could explain the longer length of the small intestine of *Arctocephalus australis*, although we hypothesize that it could be related to their food habits or, as mentioned before, a different strategy to extract food nutrients compared to the dog. Apart from these differences, the conclusion is that being a carnivore seems to be the critical characteristic defining the digestive apparatus of the *Arctocephalus australis*, the adaptations to the aquatic environment being less significant.

## Figures and Tables

**Table 1:** Morphometric data of the tongue of *Arctocephalus australis*. A: adult; J: juvenile.

Age			Length	Diameter (cm)			Weight
ID	Group	Sex	(cm)	Base	Body	Tip	(g)
M7319	A	♀	16	14	12	6	135
M99	A	♀	14	13	12	15	130
M104	J	♂	11	8.5	8	4	86
M100	J	♀	9.5	7.3	6.5	3	62.5
M74	J	♂	10	8	6.5	4.5	50
M83	J	♂	10.2	8.5	8	4.5	65
M82	J	♀	9	10	7.8	5	58.4
M70	J	♂	8.7	8	6	3	54
M79	J	♂	10.5	9.2	7	4	71
M97	J	♀	7	9	7.2	5	53.7
M84	J	♀	7.6	7.5	6	4.5	46
Mean	A		15	13.5	12	5.5	132.5
Mean	J		9.2	8.4	7	4.1	60.7

**Table 2:** Morphometric data of the *Arctocephalus australis* esophagus (11 specimens). A: adult; J: juvenile.

Age			Length	Diameter	Weight
ID	Group	Sex	(cm)	(cm)	(g)
M7319	A	♀	63	4.5	210
M99	A	♀	59	4.4	175
M104	J	♂	46	3.5	75
M100	J	♀	34	2.5	45
M74	J	♂	38	2.7	52.6
M83	J	♂	33	2.5	55
M82	J	♀	36	2.4	48
M70	J	♂	38	2.7	43
M79	J	♂	37	2.3	55
M97	J	♀	32	2	39.6
M84	J	♀	31	2.4	40
Mean	A		61	4.4	192.5
Mean	J		36.1	2.5	50.3

**Table 3:** Morphometric data of the stomach proceeding of the 11 specimens of *Arctocephalus australis* used in the present study. A: adult; J: juvenile.

Age			Length ^1^	Curvature (cm)		Diameter (cm)		Weight
ID	Group	Sex	(cm)	Major	Minor	Left Part	Pyloric Part	(g)
M7319	A	♀	60 (38 + 22)	68	42	31	17	380
M99	A	♀	47 (29 + 18)	56	41	29	15	330
M104	J	♂	32 (19 + 13)	39	21	22	10	300
M100	J	♀	27 (17 + 10)	31	20	18.5	7.5	200
M74	J	♂	28 (17 + 11)	35	17	20.5	11	164
M83	J	♂	29 (17 + 12)	32	21	17	10	190
M82	J	♀	33 (21 + 12)	42	21	23.5	13	285
M70	J	♂	32 (19 + 13)	37	22	17	10	191
M79	J	♂	33 (20 + 13)	40	19	21	11	210
M97	J	♀	37 (23 + 14)	46	24	26	14	186
M84	J	♀	32 (21 + 11)	41	21	19	12	190
Mean	A		53.5	62	41.5	30	16	355
Mean	J		31.4	38.1	20.6	20.5	10.9	212.8

^1^ Length (addition of the left and the right parts, respectively).

**Table 4:** Morphometric data of the small intestine proceeding of the 11 specimens of *Arctocephalus australis* used in the present study. A: adult; J: juvenile; D: duodenum; J: jejunum; I: ileum.

Age			Length			Diameter (cm)			Weight (g)		
ID	Group	Sex	D (cm)	J (m)	I (cm)	D	J	I	D	J	I
M7319	A	♀	43	20	16	5.5	3.7	4	25	1585	10
M99	A	♀	40	29	14	5	3.2	3.6	21	2015	12
M104	J	♂	26	15	10	4.1	3.4	2.9	16	655	6.6
M100	J	♀	19	13	15	3.5	2.4	3	13	410	8.1
M74	J	♂	22	12	9.5	3.3	2	3.2	12.5	490	6
M83	J	♂	36	13.5	6.1	4.8	2.5	2.7	15	405	5
M82	J	♀	25	14.4	7	4.5	2.6	3	19	680	4.9
M70	J	♂	27	13.5	8	4.2	2.4	2.3	12.8	525	4.3
M79	J	♂	26	14	8	3.7	2.6	3.2	17	615	4.7
M97	J	♀	22	12.5	9	2.3	2.2	2.4	11.5	765	3.8
M84	J	♀	25	12.5	8	3.5	2.5	2.6	10	405	3.8
Mean	A		41.5	24.5	15	5.2	3.4	3.8	23	1800	11
Mean	J		25.3	13.3	8.9	3.8	2.5	2.8	14	550	5.2

**Table 5:** Morphometric data of the large intestine proceeding of the 11 specimens of *Arctocephalus australis* used in the present study. A: adult; J: juvenile.

Age			Length (cm)			Diameter (cm)			Weight (g)		
ID	Group	Sex	Cecum	Colon	Rectum	Cecum	Colon	Rectum	Cecum	Colon	Rectum
M7319	A	♀	10	116	10	6	8	6	13	230	20
M99	A	♀	7	113	9	4	7	7	6	235	35
M104	J	♂	1.8	59	8.5	3.5	5.5	5.5	3.1	62	18
M100	J	♀	2.2	67	8	4	5	4.7	2.2	46	11
M74	J	♂	4.3	64	8	4.1	5.2	4.5	2.2	68	16.2
M83	J	♂	3.5	70	5.5	3.2	6	4	1.4	55	15
M82	J	♀	4	83	9	4	6.5	5.5	2	90	14
M70	J	♂	1.6	59	8	3.2	4.7	4.6	1.3	52	14.3
M79	J	♂	3	69	9	4	6	5	1.8	80	9.3
M97	J	♀	2.4	48	8	3.3	4	4.9	2.8	54	11.6
M84	J	♀	2.6	56	7	3	4.5	4.5	1.5	47.5	15.8
Mean	A		8.5	114.5	9.5	5	7.5	6.5	9.5	232.5	27.5
Mean	J		2.8	63.8	7.8	3.5	5.2	4.8	2	61.6	13.9

## Data Availability

All data supporting reported results are avalaible in Fundación Mundo Marino (by request) https://www.mundomarino.com.ar/.
